# Fluorescence Enhancement Effect of TiO_2_ Nanoparticles and Application for Photodynamic Diagnosis

**DOI:** 10.3390/ijms20153698

**Published:** 2019-07-28

**Authors:** Koki Kanehira, Yukiko Yano, Hisashi Hasumi, Hideo Fukuhara, Keiji Inoue, Kazuhiro Hanazaki, Masahiro Yao

**Affiliations:** 1 Biotechnology Group, TOTO Ltd. Research Institute, Chigasaki 253-8577, Japan; 2 Department of Urology, Graduate School of Medicine, Yokohama City University, Yokohama 236-0004, Japan; 3 Center for Photodynamic Medicine, Kochi Medical School, Kohasu, Oko, Nankoku, Kochi 783-8505, Japan; 4 Department of Urology, Kochi Medical School, Kohasu, Oko, Nankoku, Kochi 783-8505, Japan; 5 Department of Surgery, Kochi Medical School, Kohasu, Oko, Nankoku, Kochi 783-8505, Japan

**Keywords:** fluorescence enhancement effect, photodynamic diagnosis, polyethylene glycol-modified titanium dioxide nanoparticles, cellular uptake

## Abstract

Photodynamic diagnosis (PDD) can improve diagnostic accuracy by using PDD agents such as 5-aminolevulinic acid (ALA). However, the weakness and photobleaching of fluorescence of PDD agents may lead to insufficient fluorescence visibility for the detection of cancer during resection operations. We focused on the “fluorescence enhancement effect” resulting from the addition of polyethylene glycol-modified titanium dioxide nanoparticles (TiO_2_-PEG NPs) to address these problems. The results showed that the combined administration of TiO_2_-PEG NPs and ALA could enhance and prolong fluorescence in bladder cancer cells, similar to in the mixture alone. It was suggested that the fluorescence enhancement was related to the accumulation of TiO_2_-PEG NPs in cells via endocytosis, causing the light scattering and enhancement of fluorescence. This fluorescence enhancement effect could be applicable for PDD.

## 1. Introduction

Photodynamic diagnosis (PDD), a fluorescence imaging method, is widely used for the intraoperative identification of cancer tissues [1,2,3]. PDD used for non-muscle-invasive bladder cancer detection can improve diagnostic accuracy using agents such as 5-aminolevulinic acid (ALA) or hexaminolevulinate hydrochloride [4,5]. American and European guidelines recommend the use of PDD adjunctively for transurethral resection of bladder cancer using a cystoscope [6,7].

Despite the usefulness and diagnostic efficacy of PDD, the weakness and photobleaching of fluorescence may lead to insufficient fluorescence visibility for the detection of all cancer tissues during resection operations [8]. In order to overcome these issues, it is possible to use combined administration with an antioxidant to reduce the photobleaching of protoporphyrin IX (PpIX) generated from ALA [8], prevent PpIX efflux by regulation of the ATP-binding cassette sub-family G member 2 (ABCG2) transporter [9], and inhibit the reduction of the PpIX to heme by iron chelation [10].

These previous studies were limited in terms of the chemical and biological properties of PpIX. We believe that a physical approach for enhancing and prolonging the fluorescence from PDD agents can be widely used across many types of PDD agent and together with other approaches.

In the present study, we focused on the “fluorescence enhancement effect” using the light scattering properties of titanium dioxide (TiO_2_) to improve the strength and resilience of fluorescence in PDD. The goal of this study was to elucidate the effect and mechanism of enhanced fluorescence. We use polyethylene glycol-modified TiO_2_ nanoparticles (TiO_2_-PEG NPs) to inhibit aggregation and reduce their cytotoxicity, as mentioned in our previous report [11]. Using ALA as a model PDD agent, we performed validation studies on the fluorescence enhancement effect of a mixture of TiO_2_-PEG NPs and PpIX, analysis of cellular-uptake of TiO_2_-PEG NPs by bladder cancer cells, and the fluorescence enhancement effect on bladder cancer cells by the combined administration of TiO_2_-PEG NPs and ALA. 

## 2. Results

### 2.1. Properties of TiO_2_-PEG NPs and Toxicity in Bladder Cancer Cells

Nanoparticles consisting of 100 nm TiO_2_-PEG NPs, which had little cytotoxicity in terms of size-dependent apoptosis, were prepared by adapting our previous report [12]. The dispersion properties of the nanoparticles and cell viability after administration to bladder cancer cells were evaluated.

**Figure 1:**
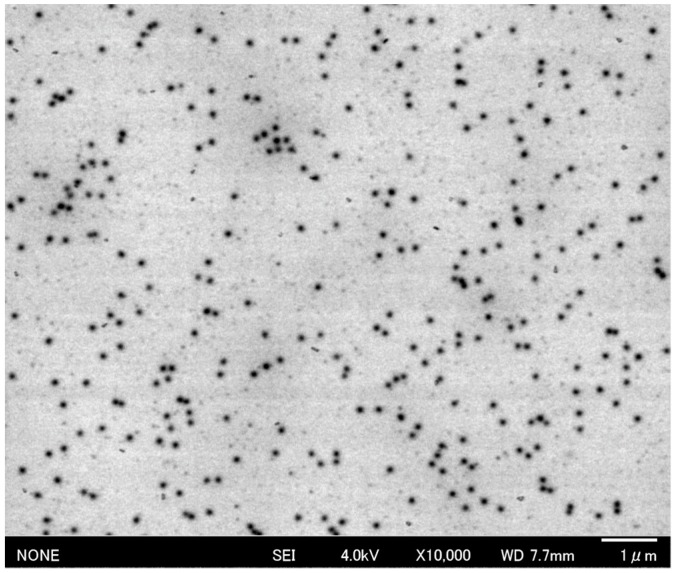
Direct scanning electron microscopic image of TiO_2_-PEG NPs.

We employed direct scanning electron microscopy (SEM) to observe the TiO_2_-PEG NPs in water as natural dispersion (Figure 1). The uniform, spherical nanoparticles were well dispersed individually and rarely aggregated. The observed diameter of nanoparticles was approximately 100 nm. To evaluate the dispersity of TiO_2_-PEG NPs, hydrodynamic particle size and zeta potential were measured by using a dynamic light scattering method. The hydrodynamic particle size of TiO_2_-PEG NPs in water was 123.8 nm. Table 1 shows the hydrodynamic particle size and zeta potential of TiO_2_-PEG NPs dispersed in minimum essential medium (MEM). The zeta potential after mixing indicated neutral, 0.17 mV at 2 h and 0.807 mV at 4 h. The sizes of the nanoparticles were almost stable in MEM medium, the nanoparticles aggregated slightly at 4 h after mixing, which was confirmed by an increase of 39.6 nm in average particle size. 

**Figure 2:**
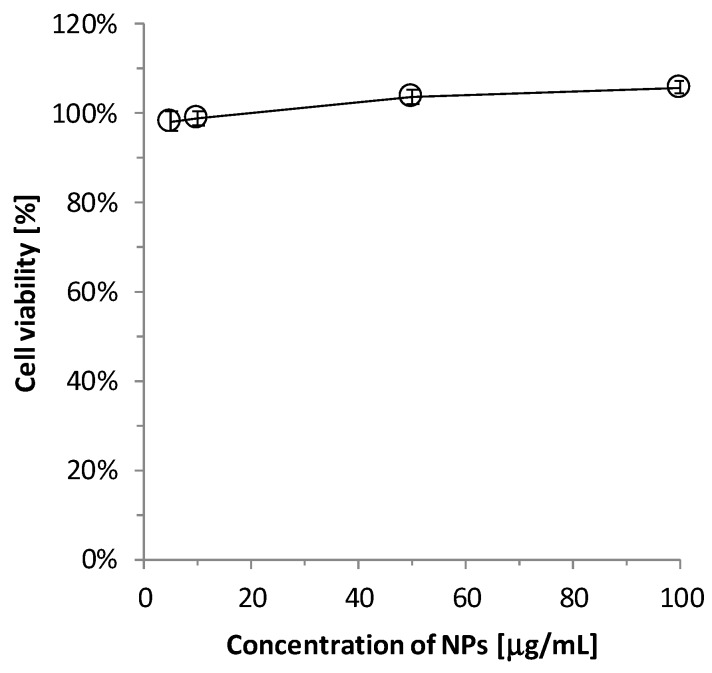
Cell viability at 4 h after TiO_2_-PEG NPs administration.

Next, the cytotoxic effect of TiO_2_-PEG NPs against UMUC3 human urinary bladder cancer cells were tested using an ATP assay method. Because UMUC3 cell line can generate relatively less amounts of intracellular PplX than other human urinary bladder cancer cells and the fluorescence is weak to visualize the cancer, we thought that UMUC3 cell line was more suitable in vitro model of bladder cancer to improve the weakness and photobleaching of the fluorescence. As shown in Figure 2, the cell viability was from 97% to 102% in the presence of 10–100 µg/mL at 4 h after administration. The results showed that the cytotoxic effects of TiO_2_-PEG NPs on UMUC3 cells were negligible in these conditions.

### 2.2. Fluorescence Enhancement Effect in a Mixture of TiO_2_-PEG NPs and PpIX

**Figure 3:**
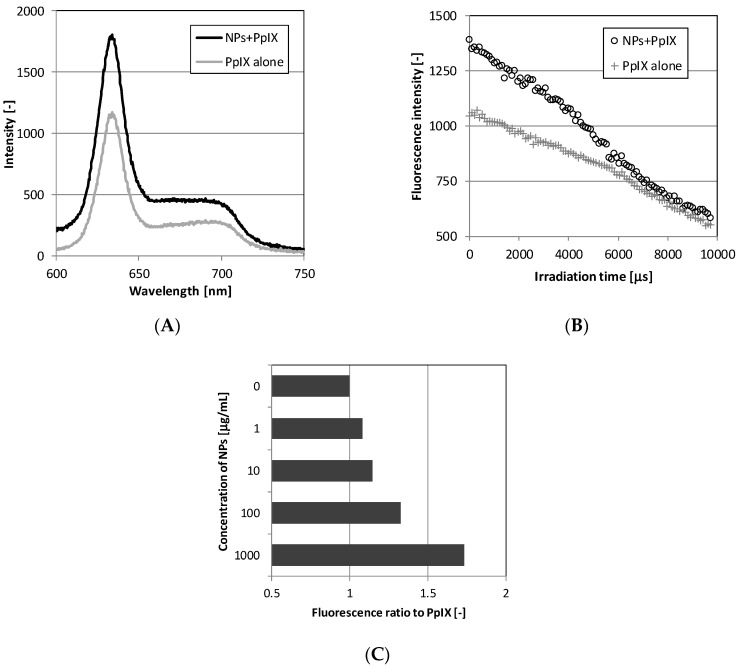
Measurement of fluorescence spectrum of the TiO_2_-PEG NPs and PpIX mixture: (**A**) Spectrum intensity of the TiO_2_-PEG NPs and PpIX mixture; (**B**) Time course of fluorescence intensity during irradiation; (**C**) Evaluation of the fluorescence ratio of TiO_2_-PEG NPs and PpIX to PpIX alone according to the concentration of TiO_2_-PEG NPs.

To confirm the effect of enhancing fluorescence from PpIX by TiO_2_-PEG NPs, the fluorescence spectrum in a solution of a mixture with 1000 µg/mL TiO_2_-PEG-NPs and 10 µM PpIX was compared with that of 10 µM PpIX alone. To model the thickness of cells, an approximately 70 µm thickness in the slide glass chambers including the sample solutions adjusted with MEM + 10% fetal bovine serum (FBS) medium were prepared for the measurements. Figure 3A indicates that the overall spectrum of the mixture of TiO_2_-PEG NPs and PpIX had higher intensity than that of PpIX in the wavelength range 600–750 nm. Both spectra had similar overall shapes, with the highest peak at 635 nm, suggested that this peak came from PpIX. 

Figure 3B shows the change in fluorescence intensity over time for both samples resulting from continuous irradiation with the excitation light source. The fluorescence intensity of the mixture of TiO_2_-PEG NPs and PpIX was higher than PpIX alone immediately after irradiation, and it maintained higher intensity with decay until 10,000 µs. The retention times of fluorescence, not less than 1000 fluorescence intensity, were 4606 µs in for TiO_2_-PEG NPs and PpIX and 1534 µs for PpIX. These results showed that TiO_2_-PEG NPs with PpIX prolonged the retention time of fluorescence 3.0-fold compared with PpIX alone. 

Furthermore, the effect of the concentration of TiO_2_-PEG NPs was examined. Figure 3C shows that the fluorescence intensity ratio compared with PpIX alone tended to increase with increasing nanoparticle concentrations in the range 1–1000 µg/mL. These results suggested that the fluorescence of PpIX was enhanced in the presence of TiO_2_-PEG NPs.

### 2.3. Cellular-Uptake Analysis of TiO_2_-PEG NPs

**Figure 4:**
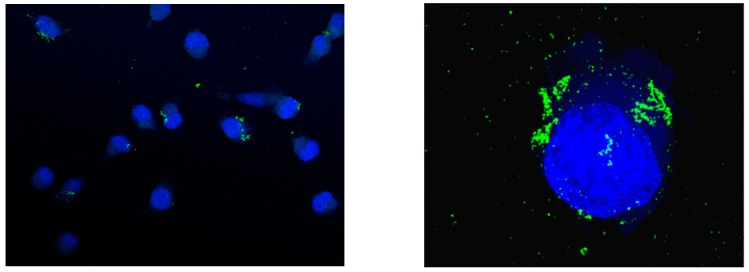
Fluorescence microscopic imaging of UMUC3 cells at 30 min after administration of fluorescently labeled TiO_2_-PEG NPs: blue indicates nuclei, green indicates fluorescently labeled TiO_2_-PEG NPs (**Left**) at 100× magnification; (**Right**) 3D imaging at 630× magnification.

The cellular-uptake behavior of fluorescently labeled TiO_2_-PEG NPs at 30 min after administration was confirmed using a super-resolution fluorescence microscopic imaging technique (HyVolution, Leica microsystems). As observed in Figure 4, the fluorescently labeled TiO_2_-PEG NPs attached to the cell surface causing flocculation, and a proportion of the nanoparticles were taken up into the cells.

**Figure 5:**
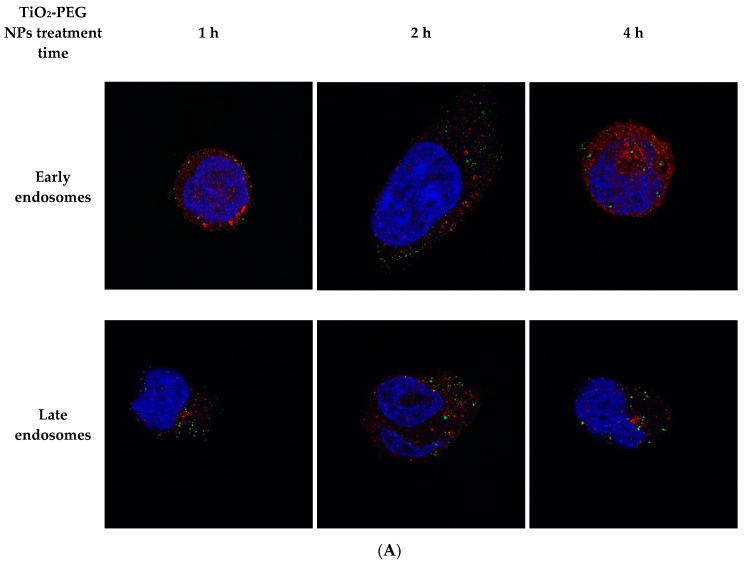

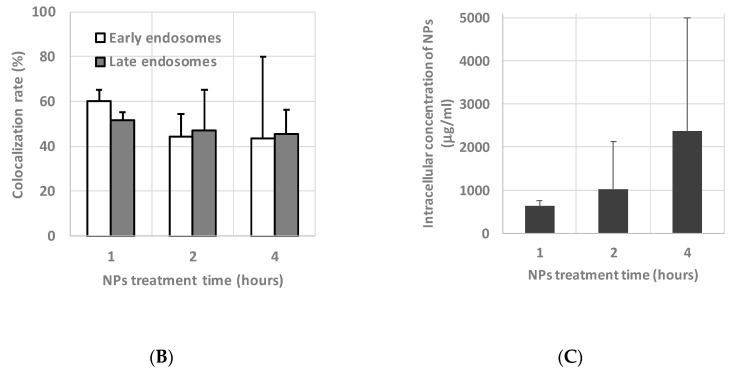
Analysis of endocytosis by UMUC3 cells after administration of fluorescently labeled TiO_2_-PEG NPs: (**A**) Immunostaining images of early endosomes using an anti-EEA1 antibody at 630× magnification, blue indicates nuclei, green indicates fluorescently labeled TiO_2_-PEG NPs, red indicates immunostained endosomes (upper) late endosome stained using an anti-LAMP1 antibody (lower); (**B**) Colocalization between endosomes and TiO_2_-PEG NPs (*n* = 3, error bar mean + SD); (**C**) Intracellular concentration of TiO_2_-PEG NPs (*n* = 3, error bar mean + SD).

In our previous report, the cellular uptake of 100 nm TiO_2_-PEG NPs was shown to be via clathrin-mediated endocytosis [12]. Accordingly, immunostaining of early and late endosomes with anti-EEA1 and anti-LAMP1 antibodies, respectively, was performed to verify the endocytosis of fluorescently labeled TiO_2_-PEG NPs. Figure 5A shows fluorescently labeled TiO_2_-PEG NPs and early and late endosomes in UMUC3 cells in all conditions. The colocalization rate between nanoparticles and endosomes was analyzed using the 3D cell imaging data (Figure 5B). The colocalization rate with early endosomes was 60% at 1 h, 44% at 2 h, and 44% at 4 h after administration. The colocalization rate with late endosomes was 52% at 1 h, 47% at 2 h, and 46% at 4 h after administration. The colocalization rates with early and late endosomes were unchanged from 2 h onward.

In addition, the intracellular concentrations of TiO_2_-PEG NPs were calculated from the 3D cell imaging data of three cells per condition. Figure 5C indicates that the intracellular concentration of nanoparticles increased incrementally: 623 µg/mL at 1 h, 1023 µg/mL at 2 h, and 2361 µg/mL at 4 h. These results showed that the cellular-uptake of TiO_2_-PEG NPs into UMUC3 cells is also related to the endocytosis process.

### 2.4. Fluorescence Enhancement Effect on Bladder Cancer Cells Using Combined Administration with TiO_2_-PEG NPs and ALA

**Figure 6:**
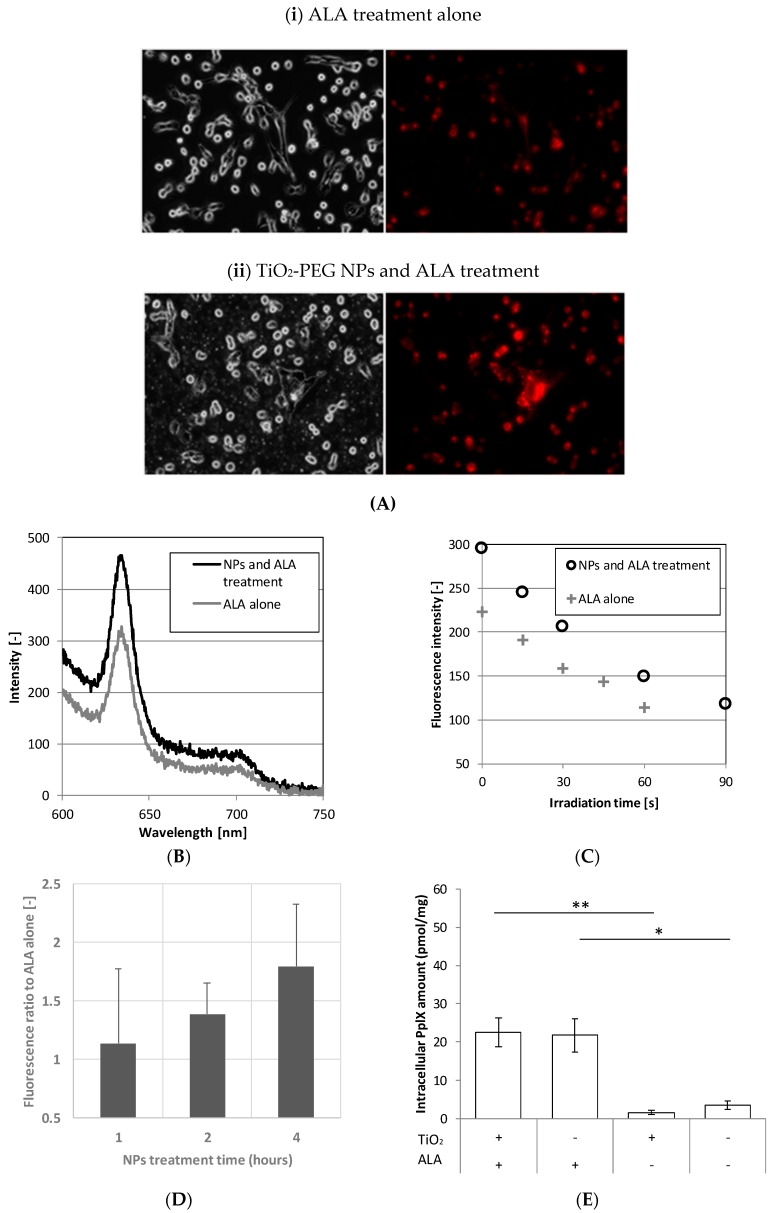
Measurement of the fluorescence spectrum in UMUC3 cells after combined administration of TiO_2_-PEG NPs and ALA: combined administration of TiO_2_-PEG NPs and ALA indicated as “NPs and ALA treatment”, administration of ALA indicated as “ALA alone”, (**A**) fluorescence microscopic imaging of UMUC3 cells at 630× magnification, administration of ALA alone ((i), upper), combined administration of TiO_2_-PEG NPs and ALA after 2 h of TiO_2_-PEG NPs treatment ((ii), lower); (**B**) Spectrum intensity of UMUC3 cells after administration of ALA alone, or combined administration of TiO_2_-PEG NPs and ALA after 2 of TiO_2_-PEG NPs treatment; (**C**) Time course of fluorescence intensity during irradiation; (**D**) Evaluation of fluorescence ratio comparing the administration of ALA alone with treatment time of TiO_2_-PEG NPs; (**E**) Intracellular amount of PpIX (*n* = 3, error bar mean ± SD, * *p* ≤ 0.05, ** *p* ≤ 0.01)**.**

To confirm the fluorescence enhancement effect of TiO_2_-PEG NPs on bladder cancer cells, we compared fluorescence between UMUC3 cells with combined administration with TiO_2_-PEG NPs and ALA and with administration of ALA alone. From the results in Figure 6A, the fluorescence image of cells treated with 10 µg/mL TiO_2_-PEG NPs and 2 mM ALA was brighter than cells treated with 2 mM ALA alone. Moreover, as shown in Figure 6B, the spectrum of combined administration with TiO_2_-PEG NPs and ALA had higher intensity than that with administration of ALA alone. Both spectra had similar overall shapes, with the highest peak at 635 nm, indicating that the peak came from PpIX. In order to verify the treatment conditions, the treatment time of TiO_2_-PEG NPs in bladder cancer cells on the combined administration for enhancement of the fluorescence was evaluated as the fluorescence intensity ratio. Figure 6C shows that the fluorescence intensity ratio was 1.1 at 1 h, 1.4 at 2 h, and a maximum value of 1.8 at 4 h.

Figure 6D shows the time course of fluorescence intensity during continuous irradiation with the excitation light source. The fluorescence intensity from the combined administration with TiO_2_-PEG NPs and ALA was higher than with ALA alone, and it was maintained at higher intensity during fluorescence decay. The retention time of fluorescence at a level of 200 or more was 37 s with the combined administration of TiO_2_-PEG NPs and ALA but only 7 s with ALA alone. Combined administration of TiO_2_-PEG NPs and ALA prolonged the retention time of fluorescence in cells was 5.3-fold higher compared with ALA alone.

Because the amount of intracellular PpIX was unclear, measurements were made with or without TiO_2_-PEG NPs. Figure 6E shows no significant difference in intracellular PpIX amount between combined administration of TiO_2_-PEG NPs and ALA and ALA alone (22.5 pmol/mg vs. 21.7 pmol/mg, *p* = 0.46). Namely, the amounts of intracellular PpIX were nearly equal whether TiO_2_-PEG NPs were administrated or not. From these results, the administration of TiO_2_-PEG NPs to cells did not affect the production of PpIX.

## 3. Discussion

We verified the fluorescence enhancement effect of TiO_2_-PEG NPs in a mixture with PpIX and nanoparticles, then confirmed the similar effect on bladder cancer cells treated with a combination of TiO_2_-PEG NPs and ALA. In addition, analysis of cellular-uptake indicated that TiO_2_-PEG NPs that were involved with the fluorescence enhancement effect accumulated intracellularly via endocytosis.

TiO_2_-PEG NPs were almost able to maintain their dispersity in medium during cell treatment. Cell viability after treatment with the nanoparticles was above 90%, showing to have little cytotoxicity. As reported on the size-dependent apoptosis effect of the nanoparticles [12], 100 nm TiO_2_-PEG NPs seemed not to have adverse effects on bladder cancer cells.

The fluorescence intensity of PpIX with TiO_2_-PEG NPs tended to increase depending on the concentration of nanoparticles. TiO_2_ has an ability to scatter light strongly because of its high reflective index, and it is used as a white pigment [13,14]. However, little was known about the fluorescence enhancement effect of TiO_2_ nanoparticles in biological conditions. The spectra with the presence and absence of TiO_2_-PEG NPs had similar overall shapes, implying that light scattering as a physical phenomena played a direct role in the fluorescence enhancement effect. The mechanism of fluorescence enhancement assumed that the scattering of nanoparticles enabled the delivery of excitation light to the fluorescent molecules effectively, and the nanoparticles also reflected fluorescence in the direction of observations. As a preliminary study, we evaluated the fluorescence enhancement effect by changing TiO_2_-PEG NPs size ranging from approximately 100 nm, 200 nm, and 300 nm at the NPs concentration of 1000 µg/mL with PpIX using the same method as described in Section 4.6. The results showed that the fluorescence intensity ratio was 1.7 of 100 nm, a maximum value of 2.0 of 200 nm, and 1.7 of 300 nm. It is well known that the optimal particle diameter for scattering light is about half the wavelength of the light. From the results of the preliminary study, we thought that the delivery of excitation light of 405 nm to the fluorescent molecules could be dominant in the fluorescence enhancement effect.

In terms of cellular uptake, we found that endocytosis by bladder cancer cells led to the accumulation of TiO_2_-PEG NPs up to high intracellular concentrations, which had the ability to enhance fluorescence. The clathrin-mediated endocytosis of TiO_2_-PEG NPs is known [12]; however, the cellular-uptake behavior of TiO_2_-PEG NPs for several hours after administration has not been clarified. Our results showed that fluorescently labeled TiO_2_-PEG NPs attached onto the UMUC3 cell surface with flocculating at 30 min after treatment. In our previous study [15], after treatment of hepatoma cells with TiO_2_-PEG NPs, flocculation of hepatocyte growth factor receptors on the cell surface was observed, which was considered to be related to the attachment of TiO_2_-PEG NPs onto cells. In the case of bladder cancer, epithelial growth factor receptor (EGFR) is overexpressed in more than 70% of bladder cancer tissue, but has a low expression in normal tissue, and UMUC3 cells also overexpress EGFR [16]. We speculated that the attachment of TiO_2_-PEG NPs to bladder cancer cells resulted in flocculating of both EGFR and TiO_2_-PEG NPs. Additionally, the colocalization rate of early and late endosomes were unchanged from 2 h onward, thus cellular uptake and the transition of nanoparticles from early endosomes to late endosomes could occur persistently. Thereby, the intracellular concentration of TiO_2_-PEG NPs was more than 100 times the administrated concentration. The data of the intracellular concentration showed some variation; therefore, individual cells might exhibit differences in nanoparticle uptake. 

Using combined administration with TiO_2_-PEG NPs and ALA, the enhanced fluorescence effect of TiO_2_-PEG NPs in bladder cancer cells was demonstrated in this study. Because UMUC3 cell line generate relatively less amount of intracellular PplX than other cancer cells shown in previous reports [17], we employed UMUC3 cell line for in vitro model of bladder cancer to improve the weakness and photobleaching of the fluorescence. A relatively low dose level of 10 µg/mL TiO_2_-PEG could enhance and prolong fluorescence. The fluorescence spectrum with the combined administration of TiO_2_-PEG NPs and ALA was similar to that of ALA alone, suggesting that the fluorescence was originally from PpIX. Additionally, the enhanced fluorescence effect had tended to increase with the treatment time with-PEG NPs. Because the amounts of intracellular PpIX were nearly equal whether TiO_2_-PEG NPs were administrated or not, the fluorescence enhancement effect is probably provided by the light scattering of intracellular TiO_2_-PEG NPs. Thus, we thought that the effects of enhancing and prolonging fluorescence resulted from the TiO_2_-PEG NPs with light scattering property accumulated close to fluorescent molecules in the cells. Furthermore, the uptake of the TiO_2_-PEG NPs by endocytosis could be the rate-limiting step of the fluorescence enhancement effect. It would be also interesting to compare the fluorescence enhancement effect between UMUC3 cells and its normal cells. However, the normal cells which have same character with UMUC3 cells are not readily available. Therefore, it was unfortunate that we could not perform the same experiment with normal cells at this time.

As shown here, this fluorescence enhancement method based on a physical mechanism of light scattering by TiO_2_ nanoparticles could be generic, so it should be possible to use it together with other PDD agents or methods for improving PDD. Additionally, the targets for this method may not only be bladder cancer but also including gastric, colon, urinary tract, and cervical cancer, where it could be applicable for PDD using endoscopy. 

This fluorescence enhancement method with TiO_2_ nanoparticles may also be applicable outside of the medical field. For example, the fluorescence method can be used for bacterial field tests in the environment studies [18]. The ability of a method to enhance and prolong the fluorescence should be useful for high-sensitivity applications such as visualizing the distribution and the types of bacterium in the field. The development of a suitable combination method with nanoparticles for other objects, such as bacteria, will assist in expanding the application of nanoparticles to other fields.

## 4. Materials and Methods

### 4.1. Preparation of TiO_2_-PEG NPs and Fluorescence Labeled TiO_2_-PEG NPs

The construct TiO_2_-PEG NPs, fluorescent labeling was performed by adapting a procedure reported previously [12]. For fluorescent labeling, 40 µmol/L of Alexa Fluor 555 NHS ester (Thermo Fisher Scientific, Waltham, MA, USA) was reacted with the same molar amount of dopamine hydrochloride (FUJIFILM Wako Pure Chemical Industries, Ltd., Osaka, Japan) in dimethylformamide (DMF) solution with 10 µmol/L of *N*,*N*-diisopropylethylamine for 3 h at room temperature. To assess the reactivity, the amount of dopamine hydrochloride before and after the reaction were measured using an HPLC-electrochemical detection system (HTEC-500, Eicom, Kyoto, Japan) in accordance with the manufacturer’s protocol. The reactivity of conjugation was more than 90%. Next, one µmol/L of the reactant from Alexa Fluor 555 with dopamine (Alexa-DA) was mixed with 0.5% (*w*/*v*) TiO_2_-PEG NPs in 20 mM HEPES buffer solution (pH 7.4) for 24 h at room temperature. The mixture was centrifuged at 14,000× *g* for 30 min and reconstituted with endotoxin-free sterilized water. This substitution process was repeated 8 times in order to remove non-reacted chemicals and organic solvents, then the fluorescently labeled TiO_2_-PEG NPs (Alexa-TiO_2_-PEG NPs) dispersions in water were collected. Fluorescence intensity of the supernatants of the Alexa-TiO_2_-PEG NPs after centrifugation was measured using a fluorescence spectrophotometer (RF-5300PC, Shimadzu, Tokyo, Japan) to determine the labeling efficiency. The labeling efficacy was more than 90% of the initial amount of mixed Alexa-DA. The evaluation of particle size and zeta potential of the nanoparticles in water and in MEM with GlutaMAX™-I and Phenol Red (42360-032, Thermo Fisher Scientific, Waltham, MA, USA) were carried out by dynamic light scattering method (zetasizer nano ZS, Malvern, UK). 

### 4.2. Direct Scanning Electron Microscopy

Direct SEM, on the basis of a frequency transmission electric-field system using SEM, was performed according to the previous report [19,20]. The sample holder with the TiO_2_-PEG NPs in water was mounted onto the sample stage. The observation conditions of SEM were captured under the following parameters: 10,000 magnifications, 1280 × 960 pixels, 160 s scanning.

### 4.3. Cell Culture

The UMUC3 human bladder cancer cell line was purchased from American Type Culture Collection (ATCC) (CRL-1749, Rockville, MD, USA). UMUC3 cells were cultured in MEM with GlutaMAX™-I and Phenol Red (42360-032, Thermo Fisher Scientific, Waltham, MA, USA) and supplemented with 10% (*v*/*v*) FBS (26140-079, Thermo Fisher Scientific, Waltham, MA, USA). Cells were cultured at 37 °C with 5% CO_2_ incubator in 100 mm culture dishes and sub-cultured every 3 days.

### 4.4. Cell Viability Assay

The cell viability of UMUC3 cells was measured using a CellTiter-Glo 2 (Promega Corp., Madison, WI, USA) in accordance with the manufacturer’s instructions. UMUC3 cells were cultured at a density of 1 × 10^4^ cells/well in a 96-well plate. After incubation at 37 °C and 5% CO_2_ overnight, the cells were exposed to TiO_2_-PEG NPs at concentrations of 0, 5, 10, 50, and 100 µg/mL. At 4 h of the exposure, the ATP content of the cells was determined using a luminoimaging camera LAS-3000mini (Fujifilm, Tokyo, Japan) after adding an equal volume of CellTiter-Glo 2 reagent to each well.

### 4.5. Fluorescence Spectrum Measurement System

**Figure A1:**
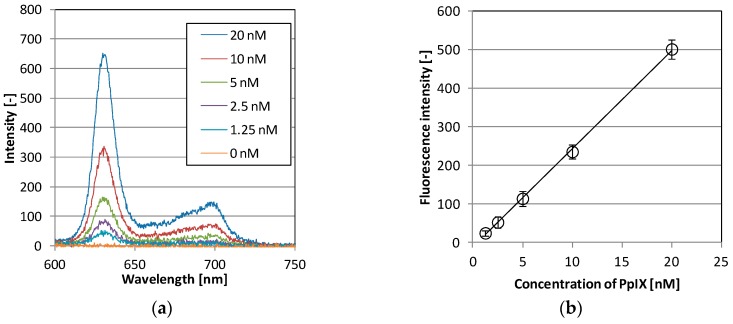
Measurement of the fluorescence spectrum of the PpIX in dimethyl sulfoxide solution: (**a**) Spectrum intensity of PpIX; (**b**) Standard curve of PpIX (*n* = 3, error bar mean ± SD).

Prior to measure the intensity of fluorescence from the objects, the measurement system was assembled. Then, 405 nm LED light (M405F1, Thorlabs, Newton, NJ, USA) and LED controller (DC4100, Thorlabs, Newton, NJ, USA) were employed as a light source. The LED was attached to a fiber optic cable (M59L0l, N.A. = 0.5, Thorlabs, Newton, NJ, USA) followed by connecting to a collimating lens (F-230SMA-A, N.A. = 0.57, Thorlabs, Newton, NJ, USA) that was vertically installed at a height of 8.0 cm from the object for irradiation. To receive the light from the irradiated objects, another fiber optic cable with a collimating lens (F-220SMA-A, N.A. = 0.25, Thorlabs, Newton, NJ, USA) that was installed at a 50° angle at a height of 8.5 cm from the object were connected to a UV-Vis spectrometer (USB2000, Ocean Optics, Dunedin, FL, USA). The spectrum data were collected and analyzed by SpectraSuite software (Version 2.0.162, Ocean Optics, Dunedin, FL, USA) under the following parameters: exposure time = 100 ms, wavelength range = 200–800 nm. The optical power of the irradiated light was 10 mW/cm^2^, monitored at a wavelength of 405 nm using a wireless power meter (PM160, Thorlabs, Newton, NJ, USA). The spectra of various concentrations of PpIX in dimethyl sulfoxide solution in cell culture plates were measured using the constructed fluorescence spectrum measurement system. Figure A1a show that all spectra derived from PpIX had a similar shape, with the highest peak at 635 nm in the wavelength range of 600–750 nm. The differential in intensity between the peak at 635 nm and background at 615 nm was defined as the fluorescence intensity of PpIX, followed by plotting the fluorescence intensity vs. concentration of PpIX to make a standard curve (Figure A1b). The correlation coefficient squared (R^2^) from the standard curve was 0.999, indicating almost perfect linearity so that the constructed fluorescence spectrum measurement system was employed for the quantitative fluorescence analysis of PpIX in cell culture plates.

### 4.6. Fluorescence Spectrum Measurement of TiO_2_-PEG Nanoparticles and PpIX Mixture

TiO_2_-PEG NPs (0, 1, 10, 100, and 1000 µg/mL) were mixed with 1 µM PpIX in MEM medium with 10% (*v*/*v*) FBS (26140-079, Thermo Fisher Scientific, Waltham, MA, USA), followed by pouring into a glass chamber plate of approximately 70 µm thickness (Sekisui Kenkyo plate UR-137-S, Sekisui Chemical, Tokyo, Japan). Each glass chamber plate was measured using the fluorescence spectrum measurement system, as described in Section 4.5.

### 4.7. Immunofluorescence Imaging

UMUC3 cells were seeded in a glass-bottomed cell culture dish at a density of 2.0 × 10^5^ cells/well in 6-well plates with fresh culture medium, then cultured overnight to allow adhesion. The culture medium was replaced by fresh culture medium with or without ALEXA-TiO_2_-PEG NPs for 0.5–4 h. Then cells were washed completely and fixed by 4% PFA for 15 min and permeabilized by 0.1% Triton X-100 for 10 min. Subsequently, the cells were blocked by 3% BSA for 30 min at room temperature and stained by anti-EEA1 antibody (ab2900, Abcam, Cambridge, MA, USA) or anti-LAMP1 antibody (ab24170, Abcam, Cambridge, MA, USA) at 4 °C overnight. After washing, cells were stained by the above secondary antibodies (Goat anti-mouse IgG (H + L) superclonal^TM^ secondary antibody, Thermo Fisher Scientific, Waltham, MA, USA) for 1 h. Finally, the stained cells were mounted by the anti-fade mounting solution (ProLong diamond antifade mounting agent with DAPI, Thermo Fisher Scientific, Waltham, MA, USA). Images were captured using a Leica SP8 confocal microscope (Leica Microsystems, Bensheim, Germany), then processed using the standard LAS X Leica software platform (Version 3.3.0.16799), and with a Leica SP8 confocal microscope and Hyvolution2 deconvolution software (Scientific Volume Imaging, Hilversum, The Netherlands).

### 4.8. Cellular Uptake Analysis

Colocalization analysis of immunofluorescence images was conducted through a LAS X Leica software platform (Version 3.3.0.16799) in accordance with the manufacturer’s protocol. The data used were respective data collected over an interval of 500 nm in the Z axis in individual cells, from 3 cells per each condition. For analysis of cellular uptake of Alexa-TiO_2_-PEG NPs to measure the size and the number, the raw data were analyzed by Python Script that functions using Anaconda (Anaconda, Inc., Austin, TX, USA) and Spyder. The results of the image analysis were further investigated using LAS X software (Leica Microsystems, Bensheim, Germany)). The amounts of Alexa-TiO_2_-PEG NPs in cells were calculated using the spherical volume from the analyzed size, and TiO_2_ material density (4.23 g/cm^3^). We assumed the shape of single tumor cell as conical, then calculated the volume of cell using their length and height from the image data taken in Section 4.7. The amounts of Alexa-TiO_2_-PEG NPs was divided by the volume of cell to determine the intracellular concentration of NPs. 

### 4.9. Fluorescence Spectrum Measurement of Human Urinary Bladder Cancer Cells Combined Administration with TiO_2_-PEG NPs and ALA

UMUC3 cells were seeded in glass-bottomed cell cultures in 24-well plates at a density of 2.0 × 10^4^ cells/well in fresh culture medium and cultured at 37 °C and 5% CO_2_ overnight to allow adhesion. After incubation overnight, the cells were exposed to 2 mM 5-ALA (FUJIFILM Wako Pure Chemical Industries, Ltd., Osaka, Japan) in culture medium for 2 h. The media were changed to TiO_2_-PEG NPs at a concentration of 10 µg/mL or MEM medium only, then cells were exposed for 1, 2, and 4 h, respectively. Each treated culture dish was washed twice with HBSS (Thermo Fisher Scientific, Waltham, MA, USA), then measured using the fluorescence spectrum measurement system as described in Section 4.5. The fluorescence ratio compared with ALA alone was calculated by the following equation.

Fluorescence ratio to ALA treatment only HBSS = (Fluorescence intensity of combined administration of TiO_2_-PEG NPs and ALA)/(Fluorescence intensity of ALA treatment only)(1)

### 4.10. Fluorescence Microscopic Imaging of Human Urinary Bladder Cancer Cells

Phase-contrast and fluorescence images were recorded using an inverted microscope (Nikon ECLIPSE Ti-E, Nikon, Kobe, Japan), then processed using the standard LAS X Leica software platform (Version 3.3.0.16799).

### 4.11. Measurement of Intracellular Amount of PpIX

Qualification of intracellular amounts of PpIX was measured as described previously [21]. To measure the protein amount in whole cells, cells were prepared as described in Section 4.9, dissolved by protein extraction reagent for mammalian cells (Apro Science, Tokushima, Japan), then the protein concentration was quantified using a protein assay BCA kit (FUJIFILM Wako Pure Chemical Industries, Ltd., Osaka, Japan) in accordance with manufacturer’s protocol. Subsequently, the preparation of a sample for PpIX measurement was performed using perchloric acid: methanol (1:1, *v*/*v*) solution. The amount of PpIX in samples was determined using a fluorescence spectrophotometer (RF-5300PC, Shimadzu, Tokyo, Japan). To measure fluorescence intensity, excitation and emission wavelengths were 405 nm and 605 nm, respectively. Intracellular amounts PpIX were calculated using the following equation.

Intracellular amount PpIX (pmol/mg) = (PpIX amount)/(Protein amount of whole cells)(2)

### 4.12. Statistical Analysis

Statistical analysis was performed with the data presented in Figure 2, Figure 5 and Figure 6. The data were displayed as mean ± SD. or mean + SD. with at least 3 parallel groups (*n* ≥ 3). The data in Figure 6 were assessed and statistically analyzed by one-way analysis of variance, * *p* ≤ 0.05, ** *p* ≤ 0.01, as shown in the figure legends.

## 5. Conclusions

**Figure 7:**
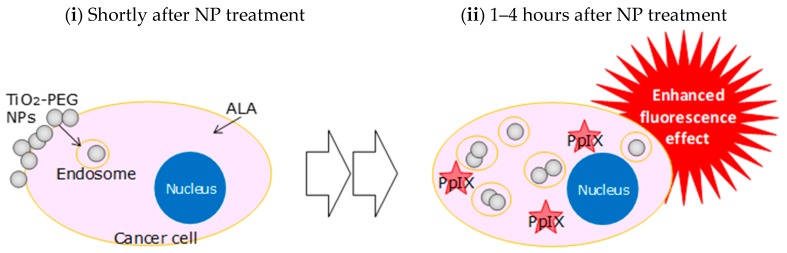
Schematic mechanism of the fluorescence enhancement effect from the combined administration of TiO_2_-PEG NPs and ALA to cancer cells. (**i**) Attachment of TiO_2_-PEG NPs to the cell membrane and continuous cellular-uptake during ALA metabolization. (**ii**) Scattering and enhancement of fluorescence from PpIX by the accumulation of TiO_2_-PEG NPs in cells.

The fluorescence enhancement effect by using TiO_2_-PEG NPs and its mechanism were elucidated. We verified that the fluorescence enhancement effect with a combination of TiO_2_-PEG NPs could improve the strength and robustness of fluorescence in PDD. As shown in Figure 7, this effect resulted from TiO_2_-PEG NPs having light scattering properties accumulated close to fluorescent molecules in cells via endocytosis processes. This fluorescence enhancement method may be applicable, not only in the medical field but also others, such as the environmental field.

## Figures and Tables

**Table 1:** Zeta potential and hydrodynamic particle size of TiO_2_-PEG NPs in cell culture medium.

Incubation Time (hours)	Average Particle Size (nm)	Zeta Potential (mV)
2	127.2	0.170
4	166.8	0.807
